# Combination of heat shock protein 90 and focal adhesion kinase inhibitors synergistically inhibits the growth of non-small cell lung cancer cells

**DOI:** 10.18632/oncoscience.245

**Published:** 2015-09-15

**Authors:** Philip J. Webber, Chanhee Park, Min Qui, Suresh S. Ramalingam, Fadlo R. Khuri, Haian Fu, Yuhong Du

**Affiliations:** ^1^ Department of Pharmacology, Emory University, Atlanta, GA, USA; ^2^ Emory Chemical Biology Discovery Center, Emory University School of Medicine, Emory University, Atlanta, GA, USA; ^3^ Department of Hematology and Medical Oncology, Winship Cancer Institute, Emory University, Atlanta, GA, USA

**Keywords:** Hsp90 inhibitor, FAK inhibitor, synergistic effect, non-small cell lung cancer (NSCLC), Akt-mTOR signaling

## Abstract

Discovery of effective drug combinations is a promising strategy to improve patient survival. This study explores the impact of heat shock protein 90 (Hsp90) inhibition in combination with focal adhesion kinase (FAK) inhibitor on the growth of non-small cell lung cancer cells (NSCLC cells). Our data show that 17-N-Allylamino-17-demethoxygeldanamycin (17-AAG), a well-studied Hsp90 inhibitor, synergized with FAK inhibitor, PF-573228, on the growth inhibition of NSCLC cells. This combination effect was confirmed using additional chemically distinct Hsp90 inhibitor, STA-9090, which is currently undergoing phase 3 clinical evaluation. Co-treatment of NSCLC cells with Hsp90 and FAK inhibitors significantly enhanced the inhibition on long-term colony formation compared to that with single agent. Inhibition of FAK exacerbated the G_2_ cell cycle arrest and annexin-V apoptotic staining induced by 17-AAG. Further mechanistic studies revealed that the combination of Hsp90 and FAK inhibitors reduced the activity of canonical proliferative and survival Akt-mTOR signaling, and increased pro-apoptotic caspase activation. Interestingly, FAK inhibition alone induced feedback activation of pro-survival Erk signaling, which was abrogated by co-treatment with Hsp90 inhibitors. Both Hsp90 and FAK inhibitors are undergoing clinical evaluation. Our studies suggest the tandem of Hsp90 and FAK inhibitors may provide an effective treatment option for NSCLC patients.

## INTRODUCTION

Lung cancer is the leading cause of death worldwide and the leading cause of cancer related deaths in the United States accounting for approximately 159,480 deaths in 2013 [1]. The 5-year survival rate of those diagnosed with lung cancer is approximately 16% despite recent advancements in therapy, necessitating the development of rapidly applicable and effective treatments for clinical use [2]. Combination therapies have been the subject of multiple recent studies due to the promise of overcoming difficulties in single treatment therapy, namely resistance and off-target effects at high dosages [3]. As such, the development of novel combinations may provide a route for better patient outcome using currently approved therapies or strategies undergoing late phase clinical evaluation. Identifying effective synergistic treatment combinations may provide the necessary options clinicians require to treat individual patients.

The chaperone Hsp90 requires the binding and hydrolysis of ATP when interacting with and folding client proteins, many of which are known contributors to disease (e.g. survivin, Akt, Hif-1a) [4-6]. Hsp90 is overexpressed in some lung cancer tissue, and lower Hsp90 expression correlates with longer survival suggesting its importance as a potential cancer therapy target [7]. Geldanamycin (GA) is an ansamycin antibiotic that binds and inhibits the ATP dependent function of Hsp90 preventing the folding of client proteins, and many inhibitors used are based on the GA structure [8-12]. Interestingly, Hsp90 inhibitors accumulate in cancerous tissue when compared to healthy control tissue [13, 14], and accumulation in disease tissue appears to be caused by an increase in active Hsp90 protein complexes in tumor tissue, which have a greater affinity for ATP and thusly inhibitor binding [15]. Cell culture and preclinical animal models of Hsp90 inhibition have been successful in attenuating growth and reducing viability of tumor cells [16, 17]; however, clinical efficacy has been limited in disease despite significant reductions in hepatotoxicity of inhibitors such as 17-AAG [18, 19]. STA-9090 is a novel non-geldanamycin second generation inhibitor currently undergoing phase 2 clinical evaluation, and STA-9090 performed better in a mastocytoma xenograft model when compared to 17-AAG suggesting a more favorable therapeutic profile [20].

Focal adhesion kinase (FAK) is a protein tyrosine kinase that acts as a critical mediator of cell adhesion, motility, and polarity. FAK is also a mediator of cell survival and has been the subject of developing cancer therapies due to its potential role in disease phenotypes. Several studies indicate increases in FAK mRNA and/or protein in tumor tissue when compared to controls [21-24], including a study of formalin fixed NSCLC and surrounding non-neoplastic tissue that identified significant increases in FAK expression in disease tissue, and FAK expression is positively correlated with later disease stage [25]. Furthermore, a study of 60 patients with acute myeloid leukemia found a correlation between the expression of activated autophosphorylated FAK and lower survival rate [26], perhaps indicating the therapeutic potential of FAK inhibition. Indeed, animal models of breast and pancreatic cancer have responded positively to inhibition of FAK autophosphorylation by Y15 [27, 28]. Multiple Phase 1 clinical studies involving different FAK inhibitors such as PF-573228 are currently underway [29].

While therapeutic agents for lung cancer treatments do exist, they lack efficacy over an extended period of time due to developing resistance and dosage limitations. Synergizing combination therapies targeting distinct molecular mechanisms in cancer may provide a means to overcome such roadblocks using existing therapeutic agents. Hsp90 and FAK are proteins that strongly contribute to disease progression, and inhibition of each protein individually was shown to reverse tumor progression in animal models. We hypothesized that concomitant targeting of FAK and Hsp90 activities may more effectively reverse tumor phenotypes compared to single inhibitor applications. Indeed, our combination screening of the NCI Developmental Therapeutics Program (DTP)'s oncology drug set revealed a positive interaction between 17-AAG and PF-573228 in lung cancer cells, in support of our hypothesis. The present study reports the discovery of synergy between Hsp90 and FAK inhibitors and mechanistic outcomes of co-inhibition of these cancer related proteins. The combination effect is associated with enhanced cell cycle arrest and apoptosis in treated NSCLCs. The effects observed in cells are linked to reduced Akt and Erk½ survival signaling. This study demonstrates that combined use of FAK and Hsp90 inhibitors synergistically antagonize the tumorigenic properties of NSCLC cells, and application of this combination with two inhibitors undergoing clinical trial may provide an effective clinical therapy.

## RESULTS

### Concomitant treatment with Hsp90 and FAK inhibitors synergistically inhibits the growth of NSCLC cells

**Figure 1:**
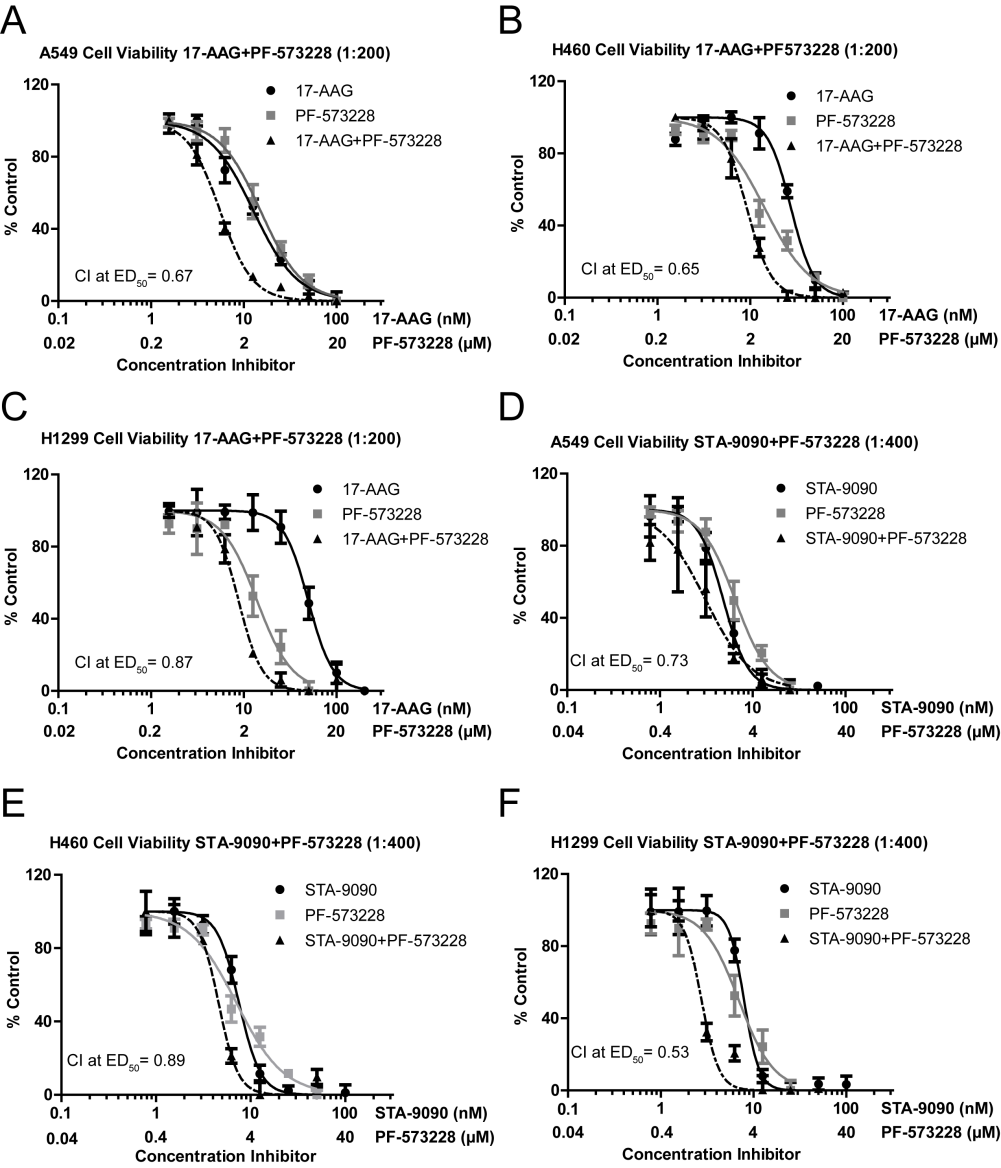
Co-treatment with Hsp90 and FAK inhibitors synergistically reduce the growth of NSCLC cells A-F) Cell viability dose-response plots representing treatment with inhibitors of Hsp90 and/or FAK for 72h at constant dose ratios in 3 NSCLC lines. Combination index at 50% the Effective Dose (CI at ED_50_) is indicated for each combination. Transformed and normalized dose-response data was assessed for IC_50_ values and significant differences were determined by the extra sum-of-squares F-test (p<.05) using a sigmoidal dose response curve fit. Representative results were selected from three different repetitions. CI values were determined using CompuSyn software. (CI<1, synergy; CI=1, additive, CI>1, antagonistic).

In order to explore the therapeutic potential for treatment with the combination of Hsp90 and FAK inhibitors, we carried out the CellTiter-Blue cell viability assay in dose-response format in three different NSCLC cell lines. H460, A549 and H1299 cells treated with Hsp90 inhibitors 17-AAG and STA-9090 showed reduced cell viability in a dose dependent manner. 17-AAG reduced cell viability in A549 (IC_50_=22.8), H460 (IC_50_=18.4), and H1299 (IC_50_=24.7) consistent with previous reports (Figure 1A-C) [30]. STA-9090 was significantly more effective in reducing cell viability with approximately 2-3 times more potency compared with 17-AAG in the NSCLCs tested (Figure 1D-F). FAK also reduced cell viability as a single agent, as shown in Fig.1. The selective FAK inhibitor, PF-573228, was effective in reducing viability of H460 (IC_50_=5.4 μM), A549 (IC_50_=4.0 μM), H1299 (IC_50_=3.82 μM) (Figure 1) [31].

Co-treatment of cells with FAK and Hsp90 inhibitors resulted in significantly enhanced inhibition of NSCLC cell viability compared to single agent treatment (Figure 1). The dose ratios approximately represent equipotency ratios (IC_50_) for Hsp90 and FAK inhibitors, so that the inhibitors contribute equally to the observed effect [32]. The combination index (CI) values at ED_50_ were <1 in all tested NSCLCs, indicating a synergistic effect for the combination of Hsp90 and FAK inhibitors [33]. The combination allows for a dose reduction of PF-573228 to a maximum of 2.3 fold and a maximum of 7.4 fold reduction in Hsp90 inhibitors at the given ratios (Table 1). These findings extend to anchorage independent H460 cultures grown in soft agar (Data not shown), where synergism between 17-AAG and PF-573228 was observed.

**Table 1:** FAK and Hsp90 Inhibitor DRI at ED_50_

	17-AAG+PF-573228 (1:200)		STA-9090+PF-573228 (1:400)	
	17-AAG	PF-573228	STA-9090	PF-573228
A549	7.40	1.86	4.17	2.02
H460	5.79	2.09	2.86	1.86
H1299	6.51	1.39	9.88	2.35

Dose Reduction Index (DRI) represents the fold reduction in drug in order to achieve the same response when used in combination when compared to single drug treatment

**Figure 2:**
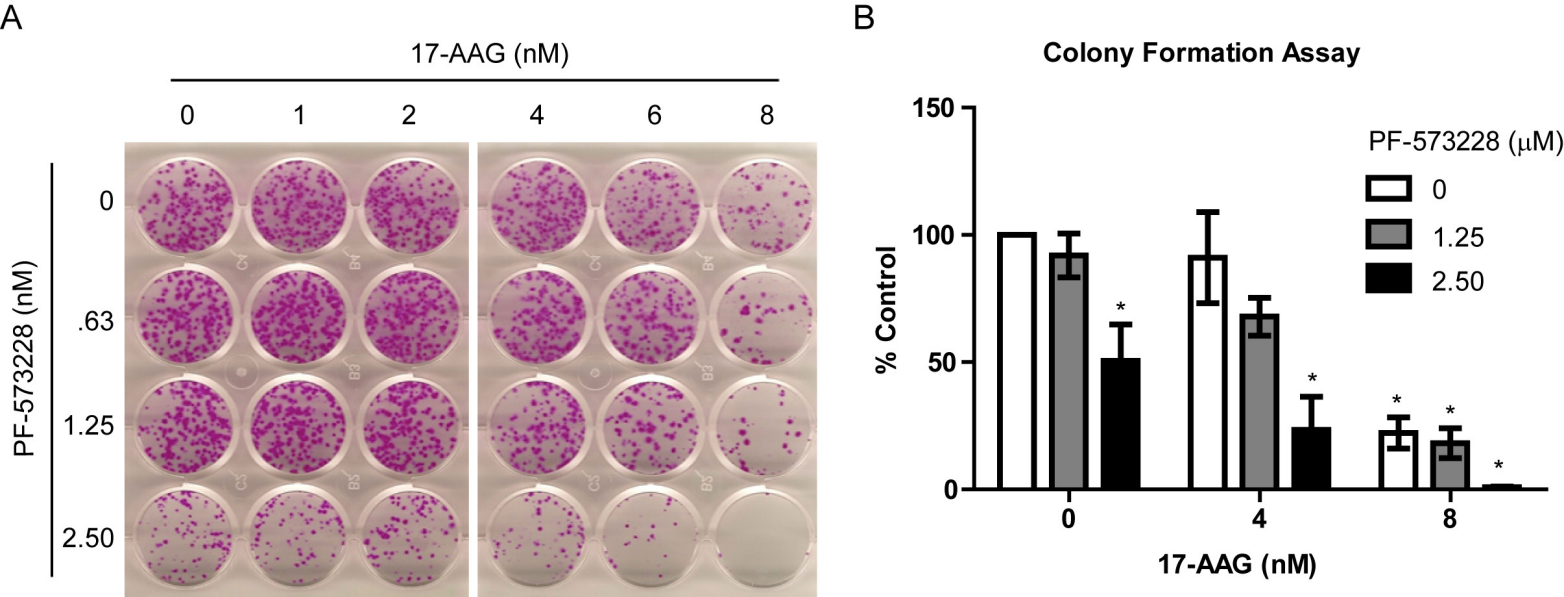
Combinations of FAK and Hsp90 inhibitors reduce long term colony formation of H460 cells A) Representative colony formation assay with indicated treatment for 10 days. B) Quantification of the colony count for 2 independent assays normalized to DMSO control. Colonies were counted with Image J and statistical analysis was conducted by one-way ANOVA with Dennett's multiple comparison post-test versus untreated control. Error bars represent SD.

Furthermore, colony formation assays indicate that H460 cells treated with FAK and Hsp90 inhibitors were exquisitely sensitive to the combination, significantly reducing the number of new colonies that formed when using as low as 4 nM 17-AAG and 1.25 μM PF-573228 (Figure 2A and B). Combination treatment effectively reduces the formation of new colonies greater than single agent strategies.

### PF-573228 enhances 17-AAG induced G_2_ cell cycle arrest and apoptosis

**Figure 3:**
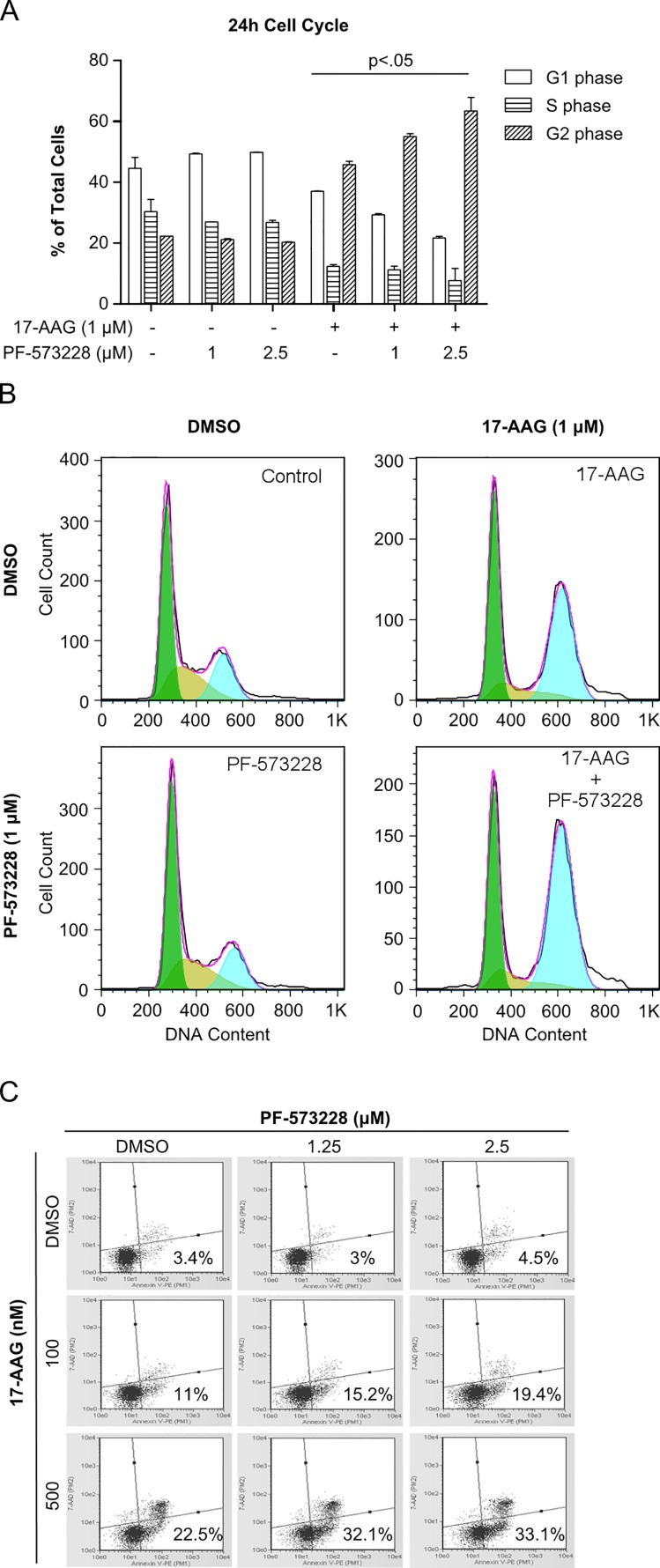
Inhibition of FAK enhances the effect of Hsp90 inhibitor on G_2_ cell cycle arrest and early apoptotic markers in H460 cells A) Cell cycle data for each phase is presented as a % of the total cells counted after 24h of treatment where data was fit to the Dean-Jett-Fox model. Analysis was conducted via two-way ANOVA with Bonferroni post-test comparing respective treated versus untreated cell cycle phase data; error bars represent SD. B) Corresponding cell cycle histograms for 24h treatment. Green=G_1/0_; Gold=S; Blue=G_2_. C) Representative histograms for annexin V and 7-AAD staining conducted 48h after treatment. Quantification of apoptotic staining is indicated for each condition.

Since the combination disrupts cell growth, we examined the cell cycle distribution of H460 cells treated with FAK and Hsp90 inhibitors. Cells were treated for 24h with compounds prior to conducting the analysis. Significant G_2_ cell cycle arrest was observed in conditions treated with 17-AAG compared to controls, while treatment with PF-573228 alone showed no significant effect at 24h (Figure 3 A and B). Interestingly, cells treated with the combination of 17-AAG and PF-573228 showed enhanced 17-AAG induced G_2_ cell cycle arrest in a dose dependent manner. At 48h, there was a marked increase in sub-G_0_ cells in treated conditions compared to controls indicating the presence of cell death, and conditions treated with the combination enhanced the sub-G_0_ population compared to single agent treatments (data not shown).

Apoptotic cells can be readily quantified with annexin V and a DNA marker that doesn't permeate intact membranes, such as 7-AAD. Interestingly, staining cells after 48h of compound treatment uncovered early apoptosis markers in cells treated with 17-AAG in a dose dependent manner, but no significant apoptotic staining was observed in cells treated with PF-573228 alone at 1.25 and 2.5 μM (Figure 3C). The combination of 17-AAG and PF-573228 resulted in a significant increase in early apoptosis, consistent with cell cycle data. These data indicate that 17-AAG induces G_2_ cell cycle arrest and apoptosis in H460 cells, and co-treatment with PF-573228 results in enhanced G_2_ phase cell cycle arrest and apoptosis.

### Co-treatment with FAK and Hsp90 inhibitors reduces canonical survival signaling, and Hsp90 inhibitors overcome FAK inhibitor-induced ERK activation

**Figure 4:**
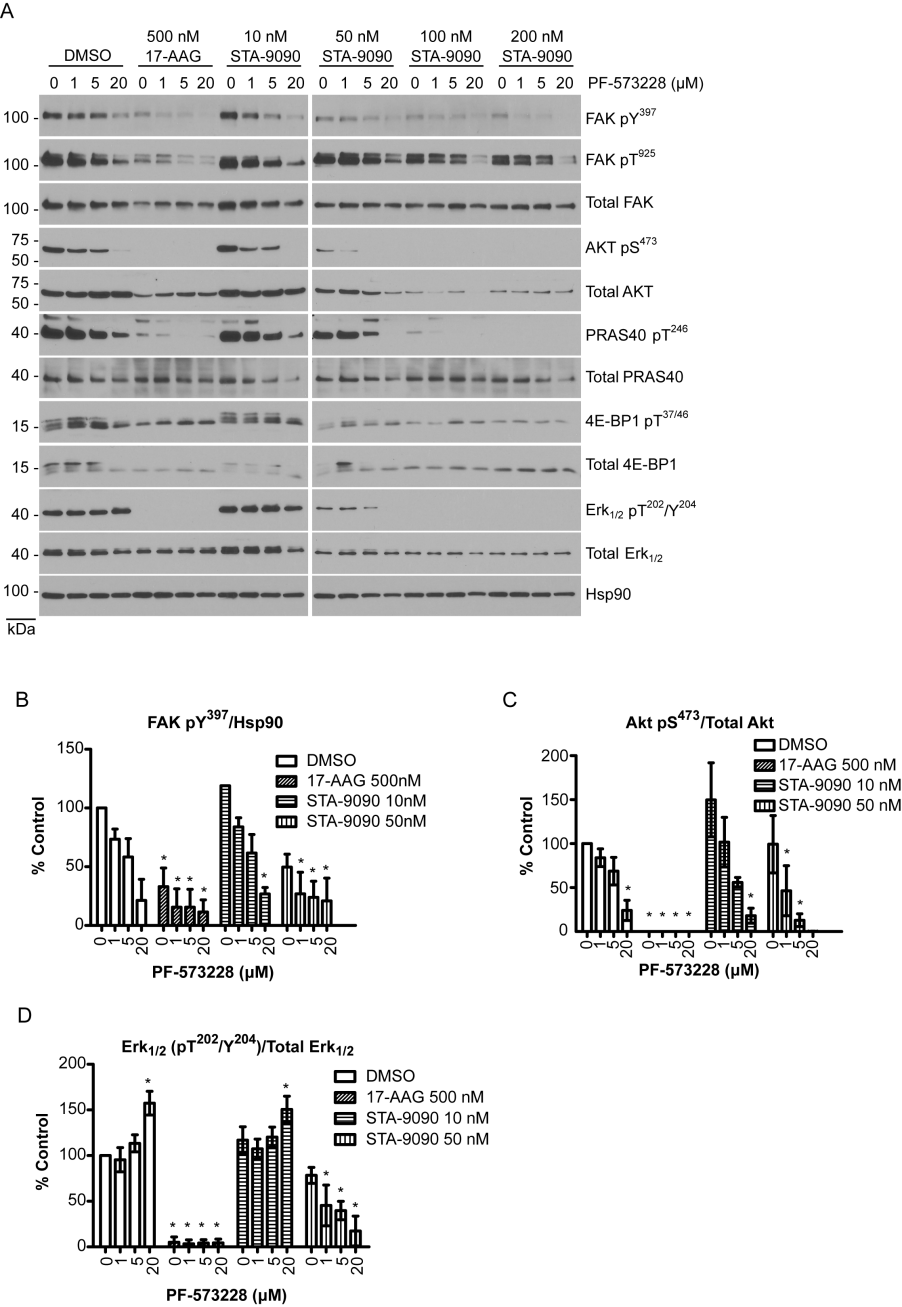
Co-treatment of Hsp90 and FAK inhibitors reduces canonical survival signaling and induces apoptotic markers in H460 cells A) H460 cells treated for 24h with Hsp90 and FAK inhibitors were used for western blot analysis of FAK, Akt and Erk½ signaling. B-D) Quantifications of western blots from A. The phosphorylated proteins, FAK pY^397^ (B), Akt pS^473^ (C), Erk½ pT^202^/Y^204^ (D), were normalized to Hsp90, total Akt and Erk½, respectively. Analyses were conducted by one-way ANOVA and Dunnett's multiple comparison post-test to compare all columns to control (* p<.05). E) Western blots were conducted for apoptotic markers after 24h of co-treatment cells with FAK and Hsp90 inhibitors alone, or in combination. Western blots are representative of 3 different experiments. Western blot densitometry quantifications are the averaged values from 3 different experiments.

The efficacy of FAK and Hsp90 inhibitors were validated using western blotting of H460 cell lysates for signaling markers after 24h of compound treatment. A trend for reduction in FAK activity was observed at 1 and 5 μM concentrations, as measured by autophosphorylation at Y397, in a dose dependent manner when cells were treated with PF-573228, and significant differences were observed at 20 μM indicating the efficacy of FAK inhibition (Figure 4A and B). Furthermore, PF-573228 (20 μM) treatment reduced the phosphorylation at FAK residue T925 by Src, which is dependent on FAK autophosphorylation at Y397. FAK protein levels and activity were also reduced in cells treated with Hsp90 inhibitors at higher doses (Figure 4A). The FAK inhibitors were more effective in reducing FAK activity when co-treated with STA-9090 at concentrations of 50 nM or greater (Figure 4A and B). Hsp90 inhibitors were especially effective at reducing the protein level of Akt, indicating that the concentrations used were effective in preventing the chaperone function of Hsp90 (Figure 4 A).

In order to determine the mechanism of action for Hsp90 and FAK target synergy, Akt/mTOR survival signaling was assessed by western blot. As shown in Figure 4A and 4C, PF-573228 and Hsp90 inhibitor co-treatment decreased the activity of Akt in H460 cells, and significant reductions in FAK activity were observed with PF-573228 1 and 5 μM when co-treated with 50 nM STA-9090. Reductions in Akt/mTOR activity were further indicated by decreased phosphorylation levels of downstream effector proteins PRAS40 and 4E-BP1 (Figure 4A).

To examine the effect of FAK and Hsp90 on additional cell survival mechanisms, we probed the status of Ras/Raf/MAP kinase pathway. Ras/Raf/Erk½ signaling plays a critical role in response to growth factors and nutrients, and aberrant activation of Ras or Raf leads to increased Erk½ activation in several different cancer types, including lung cancer [34, 35]. Unexpectedly, increased Erk½ activity was observed in H460 cells treated with FAK inhibitor PF-573228 alone (Figure 4A and D). However, PF-573228 induced Erk½ activation was not only abolished by co-treatment with Hsp90 inhibitors but resulted in decreased Erk½ activity compared to STA-9090 treatment alone (Fig. 4A, D). These data suggest that FAK inhibition alone may activate pro-survival Erk½ signaling and attenuate apoptotic signaling, but combining FAK and Hsp90 inhibition reduces Erk½ activation, resulting in further cancer cell death. Hsp90 inhibition may attenuate FAK inhibition-induced negative feedback activation on cell survival signaling, thereby accounting for the synergistic effect of the combination.

### Hsp90 and FAK inhibitors stimulate caspase 3 cleavage and reduce BAD phosphorylation

**Figure 5:**
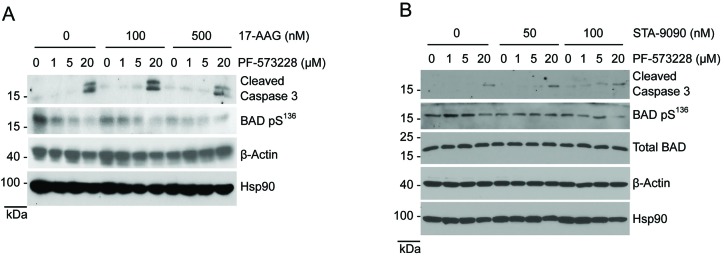
Co-treatment with Hsp90 and FAK inhibitors increases apoptotic markers in H460 cells Cells treated for 24 hours with PF-573228, 17-AAG (A) and STA-9090 (B) were stained for phosphorylated BAD pS^127^ and cleaved caspase 3.

In order to determine if decreases in Akt activity contribute to apoptotic signaling, we examined the level of Bad phosphorylation and cleaved caspase 3 after treatment of H460 cells for 24 hours with compounds. S136 phosphorylated BAD protein was reduced in response to FAK and Hsp90 inhibition (Figure 5A and B), indicating possible pro-apoptotic activity. Interestingly, cleaved caspase 3 was indeed evident when cells were treated with PF-573228 for 24 hours. These data suggest that FAK inhibitors antagonize the activity in the PI3K/Akt network and increase apoptotic signaling by alleviating suppression of BAD leading to increased caspase 3 cleavage.

## DISCUSSION

Hsp90 and FAK proteins hold potential as potent anti-cancer targets, and the expression and activity of each is correlated with cancer [7, 21-24]. Our results here show the synergistic effect of combined treatment of NSCLC cells with FAK inhibitor and Hsp90 inhibitors on inhibiting the growth of lung cancer cells. These findings extended to long-term colony formation assays in response to the combination treatment with Hsp90 and FAK inhibitors. Studies have reported little to no capacity for FAK inhibitors to induce cell death at 1 μM in cell culture models [31]. Interestingly, while consistent with previous reports, our data indicate that microMolar quantities of FAK inhibitor can be reduced by greater than 50% when combined with Hsp90 inhibitors in order to achieve the same effect on inhibiting NSCLC cell growth.

The efficacy of Hsp90 inhibitors has been previously hamstrung by gastric and hepatic toxicities. STA-9090 is more potent than 17-AAG at reducing tumor size without increasing toxicity, which has been demonstrated in mouse xenograft models [36]. FAK inhibition shows efficacy in mouse models; however, efficacy may be limited due to the overlapping function of family member proline-rich tyrosine kinase 2 (PYK2) and kinase activity independent FAK functions in tumor cells [37]. FAK scaffolding function is independent of kinase activity and may contribute to cancer phenotypes [38, 39]. Since FAK is an Hsp90 client protein, we rationalized that inhibition of both Hsp90 and FAK may reduce all tumor associated activities of FAK in addition to suppressing distinct survival functions. Our data indicate that co-treatment with Hsp90 inhibitors and PF-573228 indeed reduced FAK protein, phosphorylation, and downstream survival signaling (Figure 4A).

Hsp90 inhibitors have been shown to induce the arrest of cell cycle at the G_1_ or G_2_ phase depending on the cell type; overexpression of FAK has been reported to accelerate the G_1_ to S phase in cells [40, 41]. Our studies here have shown that Hsp90 inhibitor 17-AAG induced significant G_2_ cell cycle arrest in H460 cells, while FAK inhibitor PF-573228 alone had no effect on cell cycle at the tested doses. These results are supported by the use of a novel Hsp90 inhibitor, FS-93, which induces G2/M arrest and apoptosis in oncogene addicted cancer cell lines[42]. However, inhibition of FAK significantly enhanced G_2_ cell cycle arrest and early apoptosis when used in combination with Hsp90 inhibitor. PF-573228 alone does not enhance annexin V staining of phosphatidylserine, consistent with the lack of effect of the compound on cell growth. These data demonstrate the synergistic effect of FAK and Hsp90 inhibition on cell cycle arrest and inducing apoptosis.

Previous studies have found limited evidence for FAK inhibition induced cell death [31]. Our data show that FAK inhibitor attenuates pro-survival Akt/mTOR signaling and reduced Akt activity is coupled with reduced suppression of the pro-apoptotic BAD protein and increased caspase signaling. Unexpectedly, we have revealed that Erk½ is activated by FAK inhibitor treatment alone; it may serve as a negative feedback mechanism to maintain cell survival in response to FAK inhibitor. Interestingly, co-treatment of NSCLC cells with FAK and Hsp90 inhibitors reduces the Erk½-mediated feedback survival signaling below that of Hsp90 inhibitors alone, in part, explaining the enhanced effect by the combination.

**Figure 6:**
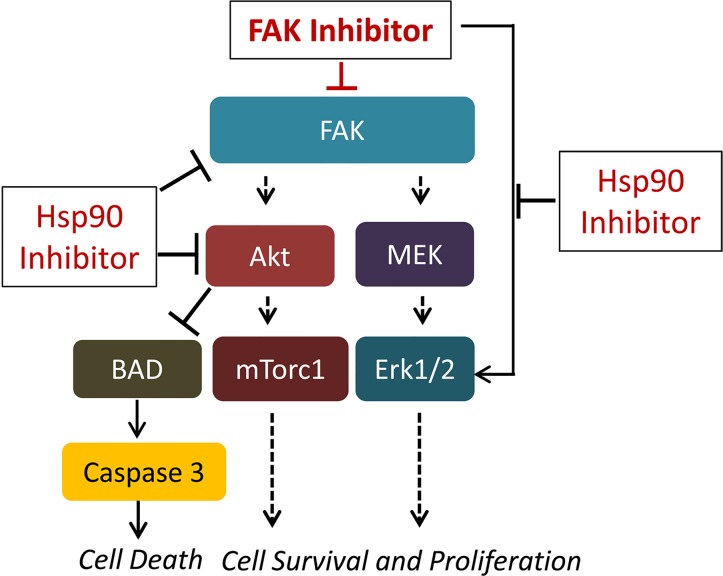
Proposed model for the combination effect of FAK and Hsp90 inhibitors Hsp90 inhibition enhances the inhibitory effect of PF-573228 on FAK/PI3K/Akt-mediated survival signaling and abolishes the FAK inhibitor-induced negative feedback activation of Erk½.

Taken together, our studies suggest that combined treatment with FAK and Hsp90 inhibitors synergistically inhibit the growth of NSCLC cells by abrogating the Akt/mTOR and Erk½ cell survival signaling, and activating caspase to induce cell death. Figure 6 depicts a model representing this proposed mechanism of action the synergistic effect of FAK and Hsp90 inhibitors. It is also interesting to note that the Hsp90 inhibitor effect could be enhanced by other means, including the activation of endoplasmic reticulum stress response[43].

The present study focuses on FAK and Hsp90 inhibitor synergy primarily in the context of cell survival and proliferation signaling and identifies a means for inducing cell death. Our observations with small molecule Hsp90 inhibitors are, in general, consistent with results from gene knockdown studies. For example, silencing of Hsp90 with shRNA inhibited Akt pathway, induced cell cycle arrest, and triggered apoptosis in breast cancer MCF7 cells [44]. On the other hand, silencing of FAK was primarily associated with reduced cancer cell migration, invasion, and metastasis [45]. It is not a surprise that FAK is a critical mediator of focal adhesion in cell attachment and migration. In addition, FAK inhibition is an established means of blocking angiogenesis, a critical step in the formation of new tumors and tumor growth [46]. Thus, it is important that this work be extended to tumor invasion and metastasis in animal models in order to determine the therapeutic extent of this synergistic combination.

The combination of FAK and Hsp90 inhibitors generates enhanced anti-cancer activity with reduced cell growth by inducing G_2_ cell cycle arrest, inhibition of pro-survival signaling, and activation of apoptosis in NSCLC cells. Inhibition of Hsp90 function by 17-AAG and STA-9090 may remove a potential resistance mechanism trigged by FAK inhibition alone. Such synergistic drug combinations provide a rapid outlet for innovative cancer treatment therapy that potentially enhances the effects of existing treatments on validated targets while reducing the dose dependent side effects from individual drugs. Further, PF-573228 and STA-9090 are currently undergoing clinical development [29]. The application of Hsp90 inhibitors along with inhibitors of FAK may provide an effective therapy for treating NSCLC patients and, potentially, broader cancer populations.

## MATERIALS AND METHODS

### Reagents

The FDA approved oncology drug set were provided by the NCI DTP (https://dtp.cancer.gov), which were used for the orthogonal screening that revealed a potential combination effect of an Hsp90 inhibitor with a FAK inhibitor. FAK inhibitors, PF-573228 and Y15, were purchased from Tocris Bioscience. Hsp90 inhibitor 17-N-Allylamino-17-demethoxygeldanamycin (17-AAG) was purchased from LC Laboratory (Woburn, MA). STA-9090 was provided by Synta Pharmaceuticals Corp. (Lexington, MA). All compounds were dissolved in DMSO as 10 mM stock and stored at −20°C. Antibodies from Cell Signaling Technologies (Danvers, MA, USA) were typically used at 1:1000 dilutions include FAK (3285), pFAK397 (3283), pFAK925 (3284), pAkt pS473 (4060), Erk½ (9102), pErk½ T202/Y204 (4370), 4E-BP1 (9452), p4E-BP1 T37/46 (9459), cleaved caspase 3 (9661). Antibodies to Akt (8312), Bad C-7 (8044), pBAD pS136 (12969), Hsp90 (13119) and β-actin (130656) were purchased from Santa Cruz Biotechnologies (USA). PRAS40 (AHO1031) and pPRAS40 pT246 (441100G) antibodies were purchased from Invitrogen (Invitrogen Corporation, CA). Secondary HRP conjugated anti-mouse and anti-rabbit antibodies were purchased from Jackson ImmunoResearch Laboratories (USA). SuperSignal West Pico Chemiluminescent Substrate (Thermo-Fisher) was used for western blot development with film. Cell culture media and Fetal Bovine Serum (FBS) were purchased from Mediatech, Inc. (Corning Cellgro, Manassas, VA).

### Cell lines and cell culture conditions

The human non-small cell lung cancer cell lines H460, A549, and H1299 were purchased from American Type Culture Collection (ATCC). The cells were cultured in RPMI-1640 media containing 10% FBS and 1% penicillin/streptomycin (Invitrogen Co., NY) at 37°C in a humidified atmosphere with 5% CO2 and 95% air.

### Cell viability assay

CellTiter-Blue cell viability assay was developed in 384-well format following manufacturer's protocol. Briefly, 1,000 cells in 45 μl cell culture media were seeded in a black 384-well cell culture plate (Corning Cat#. 3712) and incubated overnight. 0.5 μl of compound with increasing concentrations diluted in DMSO was added to the cells using Sciclone liquid handler with a 384-cannula array (Caliper LifeSciences). Vehicle (DMSO) controls were included in each plate. The final DMSO concentration was 1%. The plates were then incubated for 3 days at 37°C. 5 μl of CellTiter-Blue reagent (Promega) was then added to each well and incubated for 4h. The fluorescent intensity (FI), which is correlated with the number of viable cells, was measured using Envision multilabel plate reader (PerkinElmer) with excitation at 545 nm and emission at 615 nm.

### Colony formation assay

H460 cells were seeded at 200 cells per well in 12-well plates and incubated overnight. The cells were treated with compounds, or DMSO as vehicle control. The culture media and compounds were changed every 3 days. On day 10 the media was removed and each well was washed with phosphate buffered saline (PBS). 10% TCA was added in the each well in order to fix colonies. After washing the cells with PBS twice, the colonies were stained with 0.1% SRB (sulforhodamine B) at room temperature for 15 min as described [30]. 0.1% of acetic acid was used to wash the plates 5 times and allowed to air dry. Images were captured and the number of colonies in the each well was counted using Image J software as described [31].

### Cell cycle analysis and annexin-V staining

In a 24-well plate 1 × 10^5^ of cells/ml were seeded in each well. PF-573228 and 17-AAG were used to treat cells at indicated concentrations and time courses. Then the cells were detached with 0.25% of trypsin-EDTA and washed with serum-containing culture media. For cell cycle analysis the cells were fixed in ice-cold 70% ethanol and stored at −20°C until use. The fixed cells were harvested, washed with PBS, and stained with propidium iodide/RNase solution (Invitrogen, NY) for 15 min. The stained cells were analyzed with a Guava easyCyte flow cytometer (Millipore, MA). Cell cycle data was fit to the Dean-Jett Fox model for cell cycle analysis using FloJo software to determine the percentage of total cells in each phase. The data was then imported into GraphPad Prism for analysis. For annexin-V analysis, the trypsinized cells were washed with culture media containing FBS, stained with annexin V-PE (BioVision, USA) and 7-AAD (7-aminoactinomycin D) (AnaSpec, USA), and analyzed using Guava easyCyte flow cytometer.

### Western blotting

The cells were seeded in 12-well plates, treated with compounds at indicated doses for 24h, and then harvested with 1% NP-40 lysis buffer containing 10 mM HEPES, pH 7.4, 1 mM EDTA, 0.5% SDS, 1% NP-40, 5 mM NaVO4, 50 mM NaF, 100 mM PMSF, 1ug/mL Aprotinin and 1ug/mL Leupeptin. The cleared cell lysates were loaded and separated using 12% SDS-PAGE and transferred to PVDF membrane (Bio-Rad, USA). The membrane was probed with an antibody at 4°C for overnight. The antibodies were purchased from Cell Signaling (Millipore, MA). The developing of membrane was performed with Super Signal West Pico Substrate (Pierce; Thermo Fisher Scientific, IL). The analysis of western blotting data was performed using Image J software.

### Data analysis

Cell viability data were analyzed with GraphPad Prism 5 software (GraphPad Software, Inc.) using sigmoidal dose-response (variable slope) model. CompuSyn software (ComboSyn, Inc.) was used to calculate the Combination Index (CI) using constant dose ratios. CI<1, CI=1, and CI >1 indicate synergy, additive and antagonistic effect, respectively.
